# A narrative review of potential drug treatments for nephritis in children with IgA vasculitis (HSP)

**DOI:** 10.1007/s10067-023-06781-8

**Published:** 2023-09-27

**Authors:** Chloe E C Williams, Megan Lamond, Julien Marro, Andrew J Chetwynd, Louise Oni

**Affiliations:** 1 Royal Liverpool and Broadgreen University Hospital Trusts, Liverpool, UK; 2 Department of Women’s and Children’s Health, Institute of Life Course and Medical Sciences, University of Liverpool, Liverpool, UK; 3 School of Medicine, University of Liverpool, Liverpool, UK; 4 Centre for Proteome Research, Institute of Systems, Molecular and Integrative Biology, University of Liverpool, Liverpool, UK; 5 Department of Paediatric Nephrology, Institute in the Park Building, University of Liverpool, Alder Hey Children’s NHS Foundation Trust Hospital, Eaton Road, Liverpool, L12 2AP UK

**Keywords:** Drugs, Henoch-Schönlein purpura, IgA vasculitis, Inflammation, Kidney, Novel

## Abstract

Immunoglobulin A (IgA) vasculitis (IgAV, also known as Henoch-Schoenlein purpura, HSP) is the most common vasculitis of childhood. It usually presents with a simple, self-limiting disease course; however, a small subset of patients may develop kidney involvement (IgAV-N) which occurs 4–12 weeks after disease onset and is the biggest contributor to long-term morbidity. Treatment currently targets patients with established kidney involvement; however; there is a desire to work towards early prevention of inflammation during the window of opportunity between disease presentation and onset of significant nephritis. There are no clinical trials evaluating drugs which may prevent or halt the progression of nephritis in children with IgAV apart from the early use of corticosteroids which have no benefit. This article summarises the latest scientific evidence and clinical trials that support potential therapeutic targets for IgAV-N that are currently being developed based on the evolving understanding of the pathophysiology of IgAV-N. These span the mucosal immunity, B-cell and T-cell modulation, RAAS inhibition, and regulation of complement pathways, amongst others. Novel drugs that may be considered for use in early nephritis include TRF-budesonide; B-cell inhibiting agents including belimumab, telitacicept, blisibimod, VIS649, and BION-1301; B-cell depleting agents such as rituximab, ofatumumab, and bortezomib; sparsentan; angiotensin converting enzyme inhibitors (ACE-Is); and complement pathway inhibitors including avacopan, iptacopan, and narsoplimab. Further clinical trials, as well as pre-clinical scientific studies, are needed to identify mechanistic pathways as there may be an opportunity to prevent nephritis in this condition.

**Table:** 

**Key Points** • *Kidney involvement is the main cause of long-term morbidity and mortality in IgA vasculitis despite the current treatment recommendations.* • *The evolving understanding of the pathophysiology of IgA vasculitis is allowing exploration of novel treatment options which target underlying immune pathways.* • *Novel treatments currently being trialled in IgA nephropathy may have benefit in IgA vasculitis due to the similarities in the underlying pathophysiology, such as TRF-budesonide, B-cell modulators, and complement inhibitors.* • *Further studies, including clinical trials of novel drugs, are urgently needed to improve the long-term outcomes for children with IgA vasculitis nephritis.*

**Key Points**

• *Kidney involvement is the main cause of long-term morbidity and mortality in IgA vasculitis despite the current treatment recommendations.*

• *The evolving understanding of the pathophysiology of IgA vasculitis is allowing exploration of novel treatment options which target underlying immune pathways.*

• *Novel treatments currently being trialled in IgA nephropathy may have benefit in IgA vasculitis due to the similarities in the underlying pathophysiology, such as TRF-budesonide, B-cell modulators, and complement inhibitors.*

• *Further studies, including clinical trials of novel drugs, are urgently needed to improve the long-term outcomes for children with IgA vasculitis nephritis.*

## Introduction

Immunoglobulin A (IgA) vasculitis (IgAV, also known as Henoch-Schoenlein purpura, HSP) is a non-thrombocytopenic, small vessel vasculitis which presents acutely and most commonly in childhood. It has an incidence of 3–27 cases of IgAV per 100,000 children, characterising it as the most common paediatric form of vasculitis [1, 2]. IgAV can cause organ dysfunction affecting the skin, joints, gastrointestinal tract, and/or kidneys (termed IgAV nephritis, or IgAV-N). Excluding the renal system, manifestations of the disease are generally short-lived and straightforward to manage. IgAV-N, however, is the main contributor to long-term morbidity and mortality [3].

There is a paucity of high quality randomised controlled trials (RCTs) for the treatment of established IgAV-N and no recent data evaluating drugs which may prevent or halt the progression of early nephritis in children with IgAV. In fact, the only drug which has been evaluated in the prevention of IgAV-N is corticosteroids, which following a meta-analysis in 2009, concluded that there is no role for their use to prevent the onset of nephritis in paediatric IgAV [4–6]. Preventing the development of established kidney inflammation is a priority in children as it presents a window of opportunity to try and improve long-term outcomes. Meta-analysis of randomised controlled trials concluded that there is no role for prophylactic corticosteroids to prevent the onset of nephritis in paediatric IgAV [4]. The SHARE initiative in 2019 published consensus treatment recommendations which included the use of oral prednisolone in mild IgAV-N, with second line options including azathioprine (AZA), mycophenolate mofetil (MMF) and/or pulsed methylprednisolone and similar options, as well as cyclophosphamide (CYC) and ciclosporin, for moderate to severe disease [7]. In more specific cases such as in rapidly progressive glomerulonephritis (RPGN), Kidney Disease: Improving Global Outcomes (KDIGO) recommend managing the same as ANCA-associated vasculitis (AAV) [8]. These options, especially corticosteroids, are particularly unpopular within the paediatric population due to their side effects, with 21% experiencing weight gain, 18% having a reduction in bone density, and 19% developing Cushingoid features [9] leading to poor patient experiences and no guarantee of disease remission. Therefore, alternative therapeutic targets are needed. Therapies are being developed based on the evolving understanding of the pathophysiology of IgAV-N, which spans the mucosal immunity, kidney inflammation, complement pathway activation, and autophagy. This review article summarises potential immune targets and their stage in the therapeutic pipeline to direct future studies aimed at exploiting a window of opportunity to prevent the onset or progression of early nephritis in paediatric IgAV.

## Presentation of IgA vasculitis

### Extra-renal manifestations

IgAV is a multisystem disease which frequently presents to acute medical services with a rash, seen in up to 95% of cases at first presentation [10]. The rash tends to be erythematous, symmetrical, and petechial or purpuric in morphology. It usually follows a symmetrical distribution, and it almost exclusively originates on the lower limbs. The areas of purpura are usually palpable and non-tender and may spread to the trunk, upper limbs, and rarely to the face. Much less commonly areas of the rash can become necrotic or transform to haemorrhagic bullae, but this may be more frequently seen in adult disease.

IgAV may also affect the gastrointestinal (GI), musculoskeletal (MSK), and the kidney. GI manifestations contribute mostly to acute morbidity with colicky abdominal pain, seen in 57–96% of cases, and it is uncommonly complicated by GI bleeding, intussusception, obstruction, or GI perforation [10–12]. MSK manifestations tend to be common but short-lived; present in 47–90% of patients, typically in the form of arthralgia and/or oligoarthritis usually in the lower limb joints [10, 12–14]. This is managed conservatively as it is not known to cause any long-term erosive joint damage. Prior to the onset of the typical rash, up to 15–25% of patients report preceding joint symptoms and 10–20% report GI symptoms; however, there are no reports of kidney manifestations preceding the rash [10]. Other manifestations can include epididymitis and/or orchitis or extremely rarely, severe life-threatening manifestations such as pulmonary haemorrhage or neurological symptoms.

### Kidney manifestations

Kidney involvement is frequently asymptomatic and therefore requires active screening, with 40–50% of children having a mild, self-limiting disease course with microscopic urinary changes [14, 15]. Kidney involvement generally occurs in the first 4 weeks following diagnosis and up to 97% of cases involving the kidney will have presented by 6 months after disease onset [16]. In some children, it can present as nephrotic syndrome, nephritic syndrome, or a mix of both, and rarely as rapidly progressive glomerulonephritis [3, 11]. Potential risk factors associated with nephritis include male sex, age > 10, severe GI symptoms, arthritis/arthralgia, recurrent or persistent disease course, WBC > 15 × 10^9^/L, platelets > 500 × 10^9^/L, elevated ASO, and decreased C3 [3]. Various studies have suggested impaired kidney function, hypertension, and proteinuria at presentation, as well as key histological features including the degree of interstitial fibrosis, percentage of crescents or sclerotic glomeruli, tubular atrophy, and presence of glomeruli with fibrinoid necrosis on kidney biopsy were associated with a poorer kidney prognosis [17, 18]. By targeting the biological processes underpinning these specific factors, there may be potential opportunity to prevent or to halt the progression of significant nephritis.

## Early pathophysiological mechanisms of IgA vasculitis

**Fig. 1:**
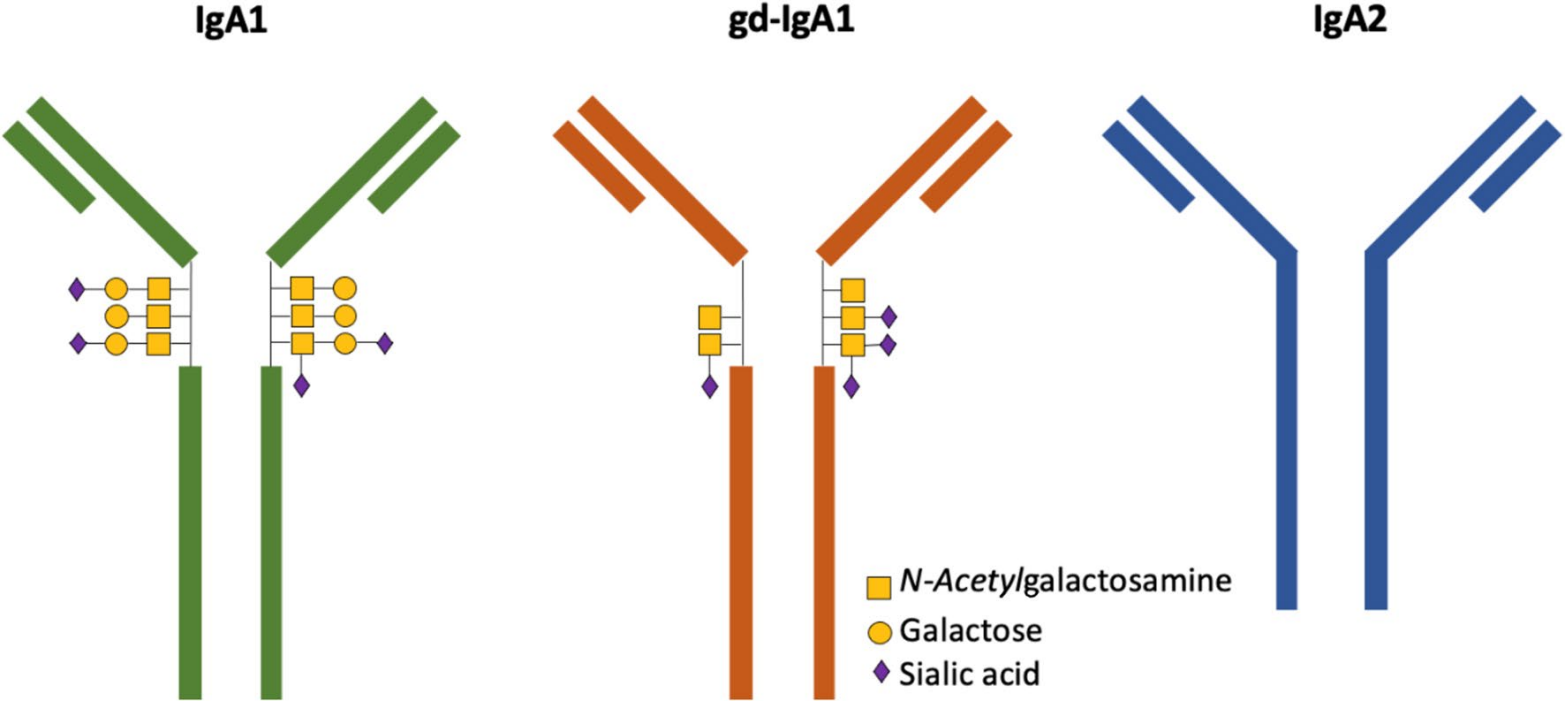
The different forms of IgA. IgA1 hinge region contains three to six O-linked glycan sites and healthy IgA1 is glycosylated with N-acetylgalactosamine, galactose, and sialic acid whereas galactose-deficient IgA1 (gd-IgA1) lacks galactose in its hinge region. IgA2 does not have a hinge region and does not possess O-glycans. Both IgA1 and IgA2 have N- glycans (not shown here). Adapted from Heineke et al. [19]

The pathophysiological mechanisms that drive IgAV are complex and incompletely understood, and many mechanistic inferences are derived from IgAN under the assumption they are related conditions. Changes to the IgA antibody are believed to direct disease progression, as IgAV patients typically present with elevated serum concentrations of galactose-deficient IgA1 (Gd-IgA1), suggesting that the O-glycans on the hinge region of IgA1 are incorrectly produced (Fig. 1). The aberrant hinge region enables diversification of structure and function acting as an epitope for anti-IgA-IgG. Consequently, this directs IgA1 immune complex formation [20]. Normal IgA is cleared via hepatocytes; however, Gd-IgA1 escapes this normal mechanism and instead deposit at vessel walls and in the mesangium inducing an inflammatory response [21].

IgAV typically follows an upper respiratory tract infection. In mucosa-associated lymphoid tissues (MALT), activation of tonsillar B-cells promotes the Gd-IgA1 profile via Toll-like receptor pathways in a T-cell-independent manner [22]. Alongside the activation of tonsillar B-cells, one paper has also identified a significantly increased concentration of total circulating B-cells in children with IgAV [23]. B cell-activating factor (BAFF, also known as B-lymphocyte stimulator, BlyS) and a proliferation-inducing ligand (APRIL) have been found to play a key role in the development of B-cells, for their survival and maturation. Elevated serum concentrations of BAFF and APRIL, as well as correlation with disease severity, have been described in multiple autoimmune diseases, including IgAN [24]. A recent study assessed whether BAFF, APRIL, and BAFFR indicated novel genetic risk factors for IgAV; however, the study demonstrated no significant differences in the genotypes between 386 IgAV patients and 806 matched healthy controls [25].

There is a potential role for anti-endothelial cell antibodies (AECAs) in IgAV, invading pathogens may have a similar antigenic structure to human vessel walls resulting in the production of cross-reactive IgA1-AECAs. Binding of IgA1-AECAs to autoantigens on endothelial cells, enhanced by TNF-α, may result in IL-8 release from endothelial cells. IL-8 can induce neutrophil migration and the interaction between IgA1-AECAs and FcαRI on neutrophils induces leukotriene B4 (LTB4) release, which results in a positive feedback loop further recruiting neutrophils. In addition, ACEAs may upregulate the expression of adhesion molecules by endothelial cells, such as E-selectin or intercellular adhesion molecule 1 (ICAM-1). Vascular damage may therefore be promoted by IgA through antibody-dependent cellular cytotoxicity (ADCC), reactive oxygen species (ROS) production, complement dependent toxicity, and neutrophils extracellular traps (NETs) formation. This process could be responsible for the characteristic leukocytoclastic vasculitis (i.e. perivascular neutrophils infiltration) seen in IgAV [19, 26].

### Pathophysiology of nephritis

**Fig. 2:**
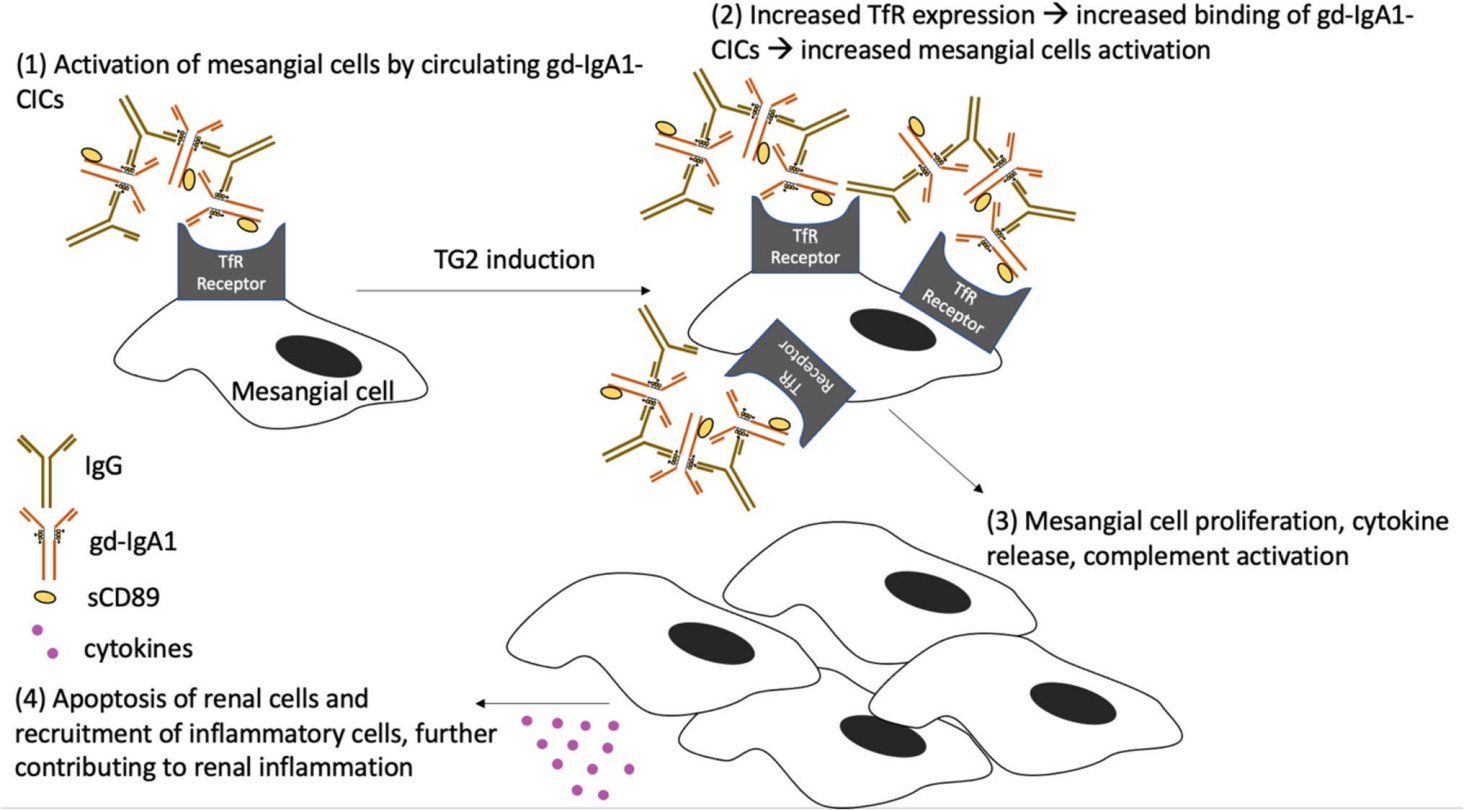
Deposition of gd-IgA1 immune complexes in the kidneys, which results in kidney inflammation. Gd-IgA1-CICs, galactose deficient IgA1 containing immune complexes; TfR, transferrin; TG2, transglutaminase 2; sCD89, soluble IgA Fc alpha receptor. Adapted from Heineke et al. [19]

Due to overwhelming systemic Gd-IgA1, kidney involvement is thought to be initiated by Gd-IgA1 complex deposition in the glomerulus which can bind to extracellularly expressed transferrin receptors (TfR) on mesangial cells [22]. Hypogalactocylation, in conjunction with the large molecular size of the Gd-IgA immune complexes, likely causes this enhanced affinity [27]. A positive feedback loop is created whereby TfR expression increases to promote downstream induction of transglutaminase 2 on the surface of the mesangium, further promoting Gd-IgA1 deposition (Fig. 2). The circulating Gd-IgA1 may contribute to mesangial proliferation, as serum IgA concentration has been demonstrated to correlate with the degree of proliferation seen in early kidney biopsies [28]. Generally, proinflammatory cytokines (IL-1β, IL-6, IL-8, IL-17A, TNF-α, IFN-γ) and adaptive immunity cytokine IL-4 serum levels are elevated whereas anti-inflammatory cytokine IL-10 levels are reduced [22, 29]. Together with cytokine production and complement activation, followed by apoptosis of kidney cells, kidney inflammation occurs alongside impairment of kidney function [19].

Endothelin-1 (ET-1) is a potent renal vasoconstrictor produced by endothelial cells which activates endothelin A receptors. ET-1 has been strongly implicated in podocyte injury, proteinuria, inflammation, and fibrosis in chronic kidney disease [30]. One study concluded plasma ET-1 concentrations were significantly raised in patients with active IgAV compared to those in remission, and a further study has demonstrated urinary ET-1 as a predictor of the duration of acute disease, suggesting the role of ET-1 is implicated in active IgAV [31, 32]. ET-1 is implicated in the renin-angiotensin-aldosterone system (RAAS) by directly stimulating aldosterone production; however, tissue necrosis factor-α (TNF-α) is also reported to increase ET-1 production [31]. These factors likely contribute to the impairment of kidney function.

#### Similarities with IgA nephropathy

IgA nephropathy (IgAN) is the most common primary glomerular disorder worldwide and is the main cause of end stage kidney failure in adults. Like IgAV, it is often preceded by an upper respiratory tract infection and usually presents with haematuria, occasionally alongside hypertension, and otherwise generally unimpressive clinical features [33]. IgAV has a significantly higher prevalence in children, with adult cases Mesangial IgA deposition seen on kidney biopsy in IgAN is indistinguishable from those with IgAV-N and consensus amongst experts accepts the two diseases as an extension of each other [34]. Underlying pathological mechanisms involving abnormal O-glycosylation, IgA1 immune complex creation, and glomerular deposition as well as involvement of the complement cascade and T-cell independent mechanisms for mucosal B-cell activation appear to be consistent between IgAN and IgAV [35–37]. Therefore, drugs which target these processes being trialled in IgAN may also have benefit in IgAV-N.

## Potential future targets

Identifying suitable drug targets presents enormous challenges for this condition as it predominantly involves children and there is a high chance of spontaneous remission (Table 1). Risk stratification of patients is therefore required, and any suitable agent should be cost effective, made in an acceptable formulation suitable for young children, and with a low risk of adverse events (AEs).

**Table 1:** A summary table of the current clinical trials internationally recruiting patients in IgA vasculitis nephritis. Data from ClinicalTrials.gov

Drug name (NCT number)	Biological target	Phase/status	Inclusion criteria	Intervention/ treatment	Primary outcome measures
Rituximab (NCT05329090)	Anti-CD20 monoclonal antibody	III/recruiting	Adults > 18 years with newly diagnosed or relapsing IgAV, must be biopsy-proven, with severe involvement of at least one organ	Rituximab plus glucocorticoids vs placebo plus glucocorticoids	The proportion of patients alive who achieved remission with a prednisone dose of 0 mg/day at both days 180 and 360
Rituximab/infliximab/tocilizumab (NCT05168475)	Anti-CD20 monoclonal antibody/TNF-α inhibitor/IL-6 receptor antagonist	II/recruiting	Age > 5 years, with a diagnosis of refractory NAAV	Patients are randomised to a sequence of up to 4 interventions: rituximab, infliximab, tocilizumab, and placebo	Time to primary treatment failure
Cyclophosphamide/MMF/leflunomide (NCT02532777)	Protein synthesis inhibitor/IMPDH inhibitor/pyrimidine synthesis inhibitor	II/unknown	Children ages 2–16 years, with kidney biopsy-proven IgAV-N and proteinuria > 50 mg/kg/day	Patients randomised to receive either cyclophosphamide. MMF or leflunomide alongside glucocorticoids and ACE-i	Disappearance of proteinuria
Benazepril (NCT02532790)	RAAS	II/unknown	Children aged 2–16 years, with kidney biopsy-proven IgAV-N	Benazepril vs prednisolone	Disappearance of proteinuria
Sparsentan (NCT05003986)	Dual-acting selective antagonist of ETA and AT1 receptors	II/recruiting	Children aged 1–18 years with kidney biopsy-confirmed disease with an eGFR ≥ 30 mL/min/1.73 m^2^	Sparsentan 800 mg in FSGS/MCD vs 400 mg in IgAN/IgAV/AS	Incidence of adverse events; u-PCR at week 108
Gamma globulin/haemoperfusion (NCT02540720)	Removal of circulating factors	II/unknown	Children aged 2–16 years with severe, dexamethasone resistant IgAV	Dexamethasone alone vs dexamethasone plus gamma globulin vs dexamethasone plus haemoperfusion	Disappearance of GI symptoms and arthralgia after 2 weeks

### Regulation of mucosal immunity

Recent evidence has pointed to mucosal antigen exposure driving disease genesis. Exposure to various environmental and/or food-related antigens is typically observed prior to disease onset [22]. The underlying mechanism remains unclear; however, one theory suggests involvement of the gut-associated lymphoid tissue (GALT). The follicle-associated epithelium, which is present on the luminal aspect of GALT, is thought to enable foreign antigens to reach the underlying immune cells, promoting their activation and ultimately the modulation of mucosal immunity [38]. Therefore, targeting mucosal immunity could form the basis for effective IgAV treatments.

#### Tonsillectomy

The tonsils have a role in regulating mucosal immunity, following antigen exposure, tonsils upregulate polymeric IgA production and in susceptible individuals with IgAV, enhance Gd-IgA production [22]. Consequently, it has been hypothesised that undergoing a tonsillectomy may enhance patient outcomes by improving the appearance of the purpura rash, alleviating abdominal pain, and reducing urine protein:creatinine ratio (UPCR) [39]. Interestingly, Japan is the only country to include tonsillectomy in their treatment recommendations for IgAV [40]. However, it is an invasive procedure and there is no convincing evidence that it is effective at treating or preventing IgAV or its associated kidney complications [11].

#### Gut microbiota

A strong correlation between intestinal microbiota and mucosal immunity is also evident. The MALT is influenced by antigenic stimulation from the commensal gut flora, and in IgAV, this is thought to influence IgA production. Evidence has demonstrated that the general abundance of intestinal microbes is lower in IgAV patients, with notable reductions in Proteobacteria and Actinobacteria [41]. At present, no trials have assessed any approaches to modulating the gut microbiome in IgAV. There is one trial currently investigating the role of faecal microbiota transplantation (FMT) in adults with IgAN (NCT05182775) with results yet to be published. There is a further study exploring the role of rifaximin in a murine model of IgAN. Rifaximin is a non-absorbable antibiotic and in this context, it aims to induce upregulation of the gut microbiota which are beneficial to the host. Whilst this study did not directly assess the gut flora, the mice who were administered rifaximin demonstrated reduced UPCR and glomerular IgA1 deposition, as well as gut BAFF and TNF-α mRNA expression [42].

#### TRF-budesonide

Recently, targeted-release formulation budesonide (TRF-budesonide) has demonstrated an ability to improve patient outcomes by targeting Peyer’s patches located within the distil ileum to decrease Gd-IgA1 levels. In a large phase IIb trial (NEFIGAN trial, NCT01738035), adults with IgAN who received TRF-budesonide demonstrated a significant reduction in proteinuria, which is indicative of a reduced risk of developing end-stage kidney disease [43]. A case report in a child with severe IgAN further demonstrated a significant reduction in UPCR with no side effects reported [44]. TRF-budesonide has not yet been trialled in IgAV; however, it may have a role as a suitable agent for early intervention in IgAV.

### B-cell modulation

#### Targeting B-cell activation

B cells are a logical early target to reduce the production of aberrant antibodies against Gd-IgA1. BAFF and APRIL are key proteins indicated in B-cell development and autoimmunity. Inhibition of BAFF alone, or dual inhibition of BAFF and APRIL, may be effective to block the binding of their receptors to prevent subsequent downstream activation of the signalling pathways associated with maintaining B-cell homeostasis [45].

Belimumab, a BLyS-neutralising monoclonal antibody, is an approved drug for the treatment of SLE. There are active trials evaluating belimumab in early SLE for prevention of LN (NCT05585671) even in paediatric patients (NCT04179032, NCT04179032, NCT04908865, NCT01649765), and emerging trials are beginning to assess its use in various vasculitides (NCT01663623, NCT03967925, NCT04629144) and glomerulonephropathies (NCT01762852, NCT01610492, NCT03949855). Unfortunately, there are no current or upcoming trials for belimumab in IgAV; however, the scientific data suggests it may have a potential role.

Several phase I, II, and III trials assessing the safety and efficacy of novel drugs promoting APRIL antagonism in IgAN have been completed recently, including VIS649 (NCT03719443) and BION-1301 (NCT03945318), which have demonstrated positive, clinically meaningful results in treating adults with IgAN. Blisibimod, a novel monoclonal antibody against BAFF, is being evaluated in both LN and IgAN across several early and late phase trials (NCT02052219, NCT02062684, NCT01395745, NCT02074020, NCT02514967, NCT01305746, NCT01162681). Despite the promise of a new therapeutic option, it is worth noting some of these trials have been terminated or withdrawn due to concerns over blisibimod’s immunogenicity and the risk of inducing neutralising antibodies that will reduce the potency of blisibimod for BAFF over time [46].

Two further novel drugs, atacicept and telitacicept, are both TACI-Ig fusion protein that are dual inhibitors of BLyS and APRIL. Atacicept has been trialled in IgAN in a phase II study (JANUS, NCT02808429), with results demonstrating dose-dependent reductions in IgA, IgG, IgM, and Gd-IgA1 and early reduction in proteinuria at week 24 in those treated with atacicept [47]. Further phase II trials continue to take place in patients with IgAN (NCT04716231, NCT02808429). Telitacicept (RC18) has been approved for the treatment of SLE in China and has had fast track approval in America for the treatment of rheumatoid arthritis. It is now being investigated in IgAN in a phase II trial with a primary outcome of change in proteinuria from baseline after 24 weeks (NCT04905212). Further pre-clinical studies are required to fully understand the role of APRIL and BAFF in IgAV before they can be assessed as a therapeutic target.

#### Targeting B-cell depletion

Rituximab (RTX) acts by binding to CD20, inducing B-cell depletion via complement- and antibody-mediated cytotoxicity [48]. The use of rituximab in refractory IgAV has been described mostly through case reports and case series with a high rate of success in achieving disease remission [49]. There are currently two trials assessing the use of rituximab in IgAV. The first is a phase III RCT evaluating rituximab versus a placebo in addition to standard glucocorticoid therapy in adults with both newly diagnosed and refractory IgAV (NCT05329090). The second is a phase II, modified crossover study which is investigating infliximab, rituximab, and tocilizumab in the treatment of non-ANCA-associated vasculitis in both adults and children of all ages with refractory vasculitis, including IgAV (“BIOVAS”, NCT05168475). Results are expected following completion in 2025 and it may give a signal to guide refractory IgAV. One of the main disadvantages to rituximab as a potential treatment for IgAV is the associated adverse events (AEs), likely rendering it unfavourable as an early preventative treatment. It is worth mentioning, however, that trials for rituximab in IgAN have demonstrated no clinical response in patients, although the reasons for this may be multifactorial [50].

There are fully humanised alternatives to RTX, for example ofatumumab (OFAB), an anti-CD20 monoclonal antibody which acts to selectively deplete B-cells. Currently licensed for the treatment of multiple sclerosis (MS), it has been used in other autoimmune diseases. A case series in 2017 demonstrated complete remission in a patient with severe IgAV-N who experienced AEs with rituximab [51]. Recent studies have shown mixed evidence regarding the efficacy of OFAB in paediatric nephrotic syndrome, focal segmental glomerulonephritis (FSGS), lupus nephritis, and post-transplant disease recurrence of FSGS; however, OFAB appears to be better tolerated than RTX [52–55].

Bortezomib (BTZ) is a proteasome inhibitor licensed for use in the treatment of multiple myeloma. It was tested *in vivo* in a murine model of lupus-like disease and demonstrated improved proteinuria, decreased autoantibody production, and prolonged survival [56]. A pre-clinical model of ANCA-associated vasculitis (AAV) further demonstrated prevention of rapidly progressive glomerulonephritis [57]. A case report was published in 2016 describing an adult case of refractory IgAV-N which was successfully treated with BTZ [58]. However, due to the high rate of peripheral neuropathy seen with BTZ treatment, it would not be desirable as an early treatment option in children.

#### SYK inhibition

Spleen tyrosine kinase (SYK) is highly expressed in primarily B-cells and myeloid cells and has a well-defined role in adaptive immune receptor signalling, a process particularly important in the initiation of inflammation. Up-regulation of SYK has been demonstrated *in vitro* in murine kidney tissue during early autoimmune vasculitis; therefore, SYK is therefore an attractive therapeutic target for inflammatory diseases, which may include IgAV [59]. An *in vivo* study revealed significant improvements in haematuria and proteinuria, as well as preservation of kidney function in a murine model of ANCA-associated vasculitis treated with 14 days of fostamatinib, the most notable SYK-inhibitor [59]. Fostamatinib has since been further investigated in a phase II randomised-controlled trial (NCT02112838) in adults with IgAN, which has shown some promising improvement in UPCR in patients with > 1 g proteinuria/day [60]. Fostamitinib, therefore, could be considered as a potential therapy in IgAV.

#### Removal of circulating complexes

One method theorised to reduce the number of circulating immune complexes that occur in IgAV is plasma exchange (PLEX). Theoretically, a high serum concentration of circulating immune complexes would be required for PLEX to be effective, and in this case, most patients would already have established nephritis. A recent systematic review of adjunct PLEX in adults with IgAV-N showed a high rate of remission (76.3%), of which 68.4% achieved complete remission and 7.8% achieved partial remission. However, 23.6% progressed to CKD stage 5 and fewer patients achieved remission if they had a transplanted kidney (20%) [61]. One study used plasma exchange in 16 children with severe, biopsy-proven IgAV-N and reported a significant increase in eGFR and reduction of urinary albumin:creatinine ratio (UACR) following 2 weeks of plasmapheresis, without the requirement for adjuvant immunosuppressive therapy [62]. Plasma exchange, however, is unlikely to have a role in early phase IgAV due to its invasiveness.

A small phase II clinical trial in China is considering the use of gamma globulin and haemoperfusion in severe IgAV (NCT02540720). The primary outcomes are improvement in GI and MSK symptoms; however, the investigators will also assess kidney function as a secondary outcome. There is no current evidence that either gamma globulin or haemoperfusion is effective treatments in IgAV-N.

### T-cell modulation

There is little evidence describing the role of T-cells in IgAV; however, emerging evidence is beginning to identify their significance in IgAN, and we may be able to extrapolate this evidence to IgAV. A recent literature review has demonstrated that in IgAN, Th2, Th17, and Tfh-type interleukins contribute to the abnormally increased production of Gd-IgA1, and that Tfh cells may stimulate the production of antibodies against Gd-IgA1 [63]. Some T-cell subpopulations have been associated with clinical severity in IgAN as well, such as higher serum interleukin (IL)-21 and IL-17A positively correlating with the severity of 24 h proteinuria and a higher frequency of Th22 cells in proteinuria-positive patients [63]. Similar findings have been observed in a small study of paediatric IgAV patients, demonstrating raised IL-17, IL-4, and IFN-gamma histologically [64]. A recent study highlighted decreased levels of circulating type 1 regulatory T (Tr1) cells, but an increased expression in the kidney tissue, as well as decreased IL-27, IL-10, and TBF-β in the serum of 50 IgAV patients. Interestingly, the results also showed a positive correlation between Tr1 cells and IL-27, perhaps suggesting the low levels of IL-27 may affect the generation of Tr1 cells, therefore downregulating downstream IL-10 and TBF-β production [65]. As IL-10 plays a key role in the resolution of inflammation, reduced levels may increase the risk of nephritis [66]. T-cells may be an appropriate immune target for treatment or prevention of nephritis; however, no specific agents are under evaluation in IgAV-N.

### Endothelin

#### ET-1 inhibition

Sparsentan is a novel, orally active drug, dual-acting as a selective antagonist of both endothelin type A (ETA) and angiotensin II type 1 (AT1) receptors [30]. A phase II clinical trial (NCT01613118), the DUET study, demonstrated a significant reduction in proteinuria following treatment with sparsentan in adults with primary FSGS when compared with irbesartan. The study also reported an extremely low rate of serious AEs (2.7%) in patients treated with sparsentan and overall good tolerability [67]. A further study, PROTECT (NCT03762850), investigated sparsentan in an international phase III double blinded study and results have demonstrated a 49.8% mean reduction in UPCR after 36 weeks of treatment, which is a threefold decrease compared to the control group who received irbesartan alone [68]. Further studies are required to assess the long-term safety and nephroprotective effects of sparsentan. Importantly, sparsentan is currently undergoing a phase II, single-arm, non-randomised cohort study (the “EPPIK” trial, NCT05003986) evaluating its safety and efficacy in paediatric patients with various proteinuric glomerular diseases including IgAV.

### Cytokines

#### Interleukin 2

Interleukin 2 (IL-2) is key in the activation of CD4 T-cells and regulates their homeostasis; it is also required for the activation in Treg cells therefore may play a role in autoimmune diseases [69]. A current clinical trial (NCT04387942) is comparing two groups of paediatric IgAV patients, the first receiving an IL-2 infusion and the second receiving traditional therapy. The outcome measures, however, do not assess the change in renal parameters and instead focus on changes in immunological responses including concentrations of circulating immune cells, immunoglobulins, and complement proteins following 7 days of treatment. Although not specifically evaluating nephritis, this trial may provide an overall indication of the efficacy of IL-2 in paediatric IgAV.

### RAAS inhibition

The RAAS acts to control blood pressure, electrolyte homeostasis, and blood volume via its effectors’ impact on the cardiovascular and renal systems. Angiotensin II regulates the secretion of aldosterone via its action on the zona glomerulosa of the adrenal glands. It also drives constriction of the post-glomerular arterioles which subsequently affects protein ultrafiltration and the further secretion of aldosterone. Angiotensin converting enzyme 1 (ACE) inhibitors (ACE-i) are well recognised for their long term renoprotective effects, and it is of a consensus opinion that patients with persisting proteinuria (i.e. > 3 months) should receive an ACE-i or angiotensin receptor blocker (ARB) to limit secondary glomerular damage [7]. A recent study demonstrated elevated urinary angiotensinogen and aldosterone in paediatric patients with IgAV when compared to healthy controls. Urinary angiotensinogen was also significantly increased when comparing those with IgAV-N to those without (IgAVwoN), suggesting the RAAS system may be activated in IgAV regardless of nephritis [70]. Results are awaited from a phase II RCT comparing the use of prednisolone with the ACE-i benazepril in children with mild proteinuric IgAV-N (NCT02532790). One study previously assessed the use of ACE-i in children with IgAN which demonstrated significant partial remission of proteinuria in those treated with ACE-i when compared to a placebo group (40.6% vs 8.8%) and total remission of 12.5% in patients treated with ACE-i compared to 0% in the placebo group [71]. Evidence from trials like these has been extrapolated to paediatric IgAV-N; hence, there has been a lack of RCTs within this population. The emerging evidence of RAAS involvement in IgAV-N highlights the use of RAAS inhibition could be further evaluated.

### Regulation of complement pathways

Emerging evidence supports complement system activation in IgAV-N, mainly through the lectin and alternative pathways [22, 72]. In one study, 90% of cases identified IgA deposits co-localised with C3 proteins within the kidney’s glomerulus and mass-spectrometric analysis demonstrated a significant increase in C5 concentration [73, 74]. Similarly, there is evidence of enhanced cleavage of the C3 and C5 proteins due to the increased plasma concentration of C3a/5a in patients with IgAV [75]. Factor D and factor B are particularly interesting complement components due to their role in the amplification loop, which alone accounts for up to 80% of complement activation [76]. Clinical trials are underway to evaluate selective complement inhibitors in adults with IgAN, with therapeutic targeting including upstream complement factors (factor B, factor D, MASP-2) as well as downstream ones (C3, C5, C5a). These include the complement inhibitors avacopan, iptacopan, and narsoplimab which have shown promising results; however; they are yet to be trialled on patients with IgAV [77–79]. There is only one case report of the use of a narsoplimab in a young woman with rapidly progressive IgAVN which resulted in sustained reduction in lectin pathway activation and a delayed decline in kidney function [80].

### Other

There is some evidence supporting the use of leflunomide (LEF) as a treatment for IgAV. One small case series demonstrated successful treatment in 5 paediatric IgAV patients with nephrotic-range proteinuria who were treated with leflunomide alongside corticosteroids [81]. A further study has shown better renal outcomes in adults treated with corticosteroids and LEF compared to those without [82]. Robust evidence is still lacking; however, LEF is included in a Chinese RCT of IgAV-N (NCT02532777) with results expected soon.

Hydroxychloroquine (HCQ) is an anti-malarial drug that was repurposed as a disease modifying antirheumatic drug (DMARD) and widely used in SLE. There have been no formal studies assessing HCQ in IgAV; however; it reduces proteinuria in lupus nephritis [83].

## Conclusion

IgAV is a common vasculitis of childhood which is mostly self-limiting; however; there is a subset of children who experience nephritis and may develop serious, long-term morbidity progressing to CKD. Globally, there are very few clinical trials taking place, and none which evaluates the use of potential early targets to prevent nephritis. Potential drugs that could be used as early targets to prevent progression of nephritis warranting further evaluation include TRF-budesonide; belimumab, telitacicept, blisibimod, VIS649, and BION-1301; rituximab and ofatumumab; sparsentan; ACE-Is; and complement pathway inhibitors including avacopan, iptacopan, and narsoplimab. Further pre-clinical scientific data are urgently needed to inform this mechanistic disease pathway.

## Data Availability

The data used to support the findings of this study are included within the article.
